# Testing Bioimpedance to Estimate Body Fat Percentage across Different Hip and Waist Circumferences

**DOI:** 10.1155/2019/7624253

**Published:** 2019-06-11

**Authors:** Viseth Long, Matthew Short, Spencer Smith, Martin Sénéchal, Danielle R. Bouchard

**Affiliations:** ^1^Cardiometabolic Exercise & Lifestyle Laboratory, University of New Brunswick, 90 Mackay Drive, Fredericton, New Brunswick, Canada E3B 5A3; ^2^Faculty of Kinesiology, University of New Brunswick, 90 Mackay Drive, Fredericton, New Brunswick, Canada E3B 5A3

## Abstract

Many studies have validated the use of bioimpedance analysis (BIA) to quantify body fat percentage (BF%). However, it is unknown if some model types (i.e., hand to hand, foot to foot, and hand to foot) are differing in their validity depending on hip and waist circumferences. The purpose of this study was to compare the difference in BF% between three BIA models (i.e., hand to hand, foot to foot, and hand to foot) against the Bod Pod across different hip and waist circumferences. A total of 92 people aged 19-72 years were recruited in this study. After following the pretesting procedures recommended for BIA measures, BF% was estimated using three BIA models and the Bod Pod. Hip and waist circumferences were obtained using standard procedures and tertiles were computed. The Bland-Altman was plotted and 1-sample T-test as well as correlation between the average measure and the difference between the two measures was tested. Within the entire sample, across all BIA models, the Bland-Altman analysis showed significant difference compared to 0 and a significant difference for the proportional. However, when stratified by tertiles, the two measurements were only significant for the highest tertiles of hip and waist for all BIA apparatus (all p <0.01) and the proportional bias was nonsignificant for all tertiles and across all BIA apparatus. For the highest tertile of waist and hip, the average difference was between 1.67% and 3.29% compared with the Bod Pod estimation. In conclusion, the three BIA models offer a BF% measurement agreeing with the estimation obtained with the Bod Pod with the exception of people having a greater waist or greater hip.

## 1. Introduction

Body fat percentage (BF%) is an important variable related to health that both clinicians and the general public are interested in knowing, as it has been strongly associated with many chronic conditions [1]. Given many health risks associated with the accumulation of BF% [2–5], health care professionals often encourage their patients to lose weight to reduce BF%.

Besides dissection, it is impossible to directly measure BF%. However, various tools have been developed to estimate it. Gold standard measures such as a computed tomography scan, dual-energy X-ray (DXA), and the Bod Pod are offering a precise estimation of BF%, but they are costly and require experienced staff and a certified facility to hold the equipment [6]. Because of these restrictions, gold standard measurements of body composition are not readily available [7].

Bioelectrical impedance analyzer (BIA) is an inexpensive and practical tool based on the principle that various body components have more or less water content and thus offer a different resistance to the passage of an electrical current, generating more or less resistance [8]. Even though BIA has been extensively validated in the past and has shown high agreement with the gold standard measures [9], it is currently unknown if the difference between BIA and a gold standard measures varies across different waist and hip circumferences or types of BIA models.

Three major groups of BIA models exist: hand to hand, foot to foot, and hand to foot. Different types of BIA analysis models could potentially show less difference compared to a gold standard measure for specific waist and hip circumferences. Since FM has a greater resistance to the electrical current compared with FFM, the current may not be able to easily travel through the body to reach all body segments, thereby affecting the validity of the measurement. For example, the hand to hand models could be more accurate if one has greater waist circumference, rather than greater hip circumference. Some research has recently been completed in this area. Aldosky et al. (2018), who looked at a sample only composed of women aged 32-85 years, reported that women having a large waist circumference would be more accurate to estimate with a hand to hand BIA than a foot to foot BIA [10]. However, no gold standard measure was included in this study.

The main objective of this study was to compare the difference of estimation BF% between three BIA models (i.e., hand to hand, foot to foot, and hand to foot) against the Bod Pod across different hip and waist circumferences.

## 2. Materials and Methods

A total of 92 participants were recruited to be part of this study in a single session. Prior to the session, participants were asked to avoid consuming anything that can impact body water content [2]. Six criteria were set for participants to follow: (1) no eating in the four hours prior to experiment, (2) no vigorous exercise in the twelve hours prior to assessment, (3) no alcoholic beverages in the twelve hours prior to assessment, (4) no water consumption for the four hours prior to testing, (5) no caffeine the day of the test, and (6) to wear tight fitting clothing which can easily be removed. Anthropometric measurements were taken. These include height, weight, and circumference of the hips and waist. These were measured following standardized procedures from the American College of Sports Medicine. BMI was calculated using the following equation: weight (kg)/height (m^2^). For BIA measurements, two out of the three models required physical activity level. Physical activity level was self-reported based on the number of aerobic minutes estimated to be done in a typical week at moderate to vigorous intensity. Inactive was considered to be ≤150 minutes at moderate to vigorous intensity, active was considered as > 150-300 minutes at moderate to vigorous intensity, and very active was considered to be >300 minutes in moderate to vigorous intensity per week [3]. For each BIA measure, participants removed their shoes. Measurements were done using three different BIA scales:

(1) Hand to hand (OMRON, HBF-306, China): height, weight, age, and sex of the participant were entered. Both hands were used to hold the device at chest level while the measurement was taken.

(2) Foot-only (TANITA, BC-533, Japan): age, sex, and physical activity level of the participant were entered. Participants were asked to step on the scale and stand straight with arms touching the hips as the measurement was taken.

(3) Hand to foot (TANITA, BC-568, Japan): age, height, and physical activity level of the participant were entered. Once this was completed, participants were asked to step on the scale, stand straight, and pull the handles up to their side while the measurement was taken.

Following the BIA scale measures, participants were asked to complete a Bod Pod test to estimate BF mass based on body density. Male participants were asked to wear tight fitting shorts while female participants were asked to wear either a sport bra and spandex shorts and a swim cap to limit errors of measurement. For proper calibration, the Bod Pod had to measure weight to within ±0.005 kg for the 20 kg calibration weight and to measure the volume of the 50.12 L calibration cylinder. Two body volume measurements, each approximately 50 seconds, were taken and if the two measurements were not consistent with each other, a third measurement was taken. Thoracic gas volume (TGV) was estimated as high correlations have been observed between estimated TGV and measured TGV [11]. The Siri equation was used to estimate body composition.

### 2.1. Statistical Analyses

Descriptive statistics are reported as means ± standard deviations for participants' physical characteristics based on tertiles of waist and hip (Table 1). Differences between tertiles were tested via ANOVA or Chi-Square. The methods used to assess the accuracy between BF% estimated with BIA and BF% estimated with Bod Pod were the Bland-Altman (average and 95% confidence intervals), the one sample T-test on the difference between each BIA and Bod Pod, and correlations to test proportional bias for each tertile of hip and waist. Statistical analyses were performed on the Statistical Package for the Social Sciences version 24.0 (SPSS) (SPSS Inc., Chicago, IL, USA)

## 3. Results

General characteristics of the subjects are presented in Table 1 as a whole and per tertiles of waist circumference and tertiles of hip circumference. The average age of participants was 43.8 ± 17.5 years and 55% of participants were male. A significant difference was observed among the waist tertiles for age, BMI, and waist and hip circumferences (p<0.05). The average BMI was considered overweight: 25.5 ± 4.3 kg/m^2^. A significant difference was observed among the hip tertiles for BMI waist and hip circumferences (p<0.05).

**Figure 1:**
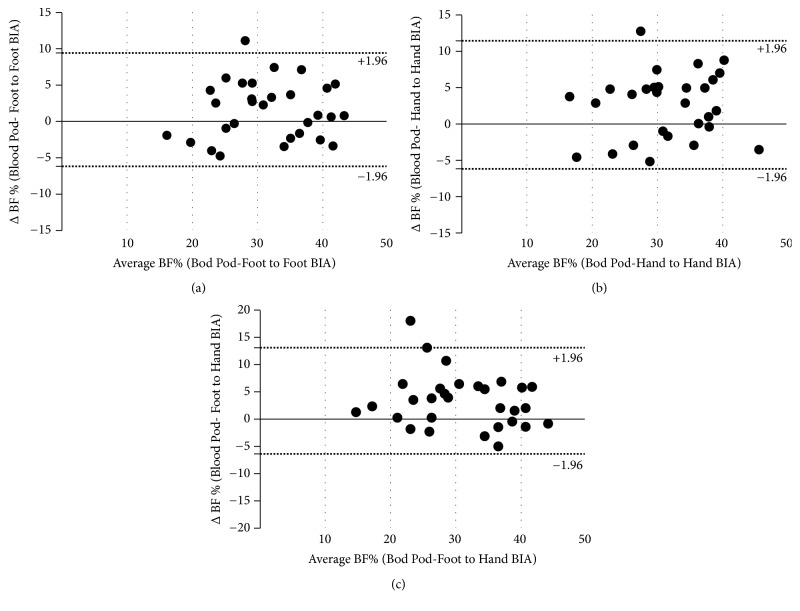
Tertile 3 of Waist Circumference Across BIA models.

**Figure 2:**
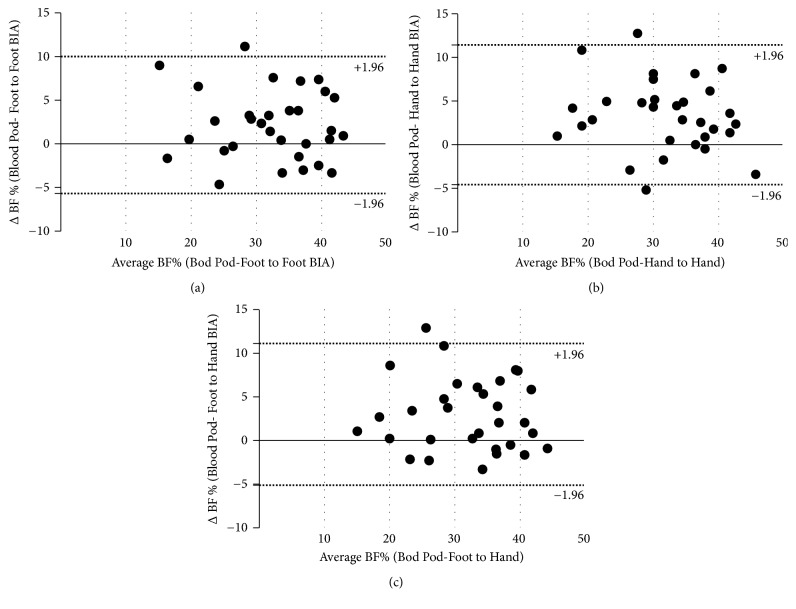
Tertile 3 of Hip Circumference Across BIA models.

The Bland-Altman method was used to plot the difference in BF% for each BIA model with the Bod Pod (i.e., Bod Pod BF% estimation minus BIA BF% estimations) against the mean of the each BIA BF% estimation with Bod Pod. Based on the results, the agreement between BIA models and Bod Pod estimations of BF% was significant for tertiles 1 and 2 whereas the highest tertiles of waist (Figures 1(a), 1(b), and 1(c)) and hip (Figures 2(a), 2(b), and 2(c)) showed the average and 95% CI. Analysis revealed a significant difference when compared with 0 difference (all p <0.01). For the highest tertile of waist and hip, the average difference was between 1.67% and 3.29% compared with the Bod Pod estimation. The proportional bias was nonsignificant for all tertiles and across all BIA apparatus.

## 4. Discussion

This study aimed to compare the accuracy of BF % between three BIA models (hand to hand, foot to foot, and hand to foot) against the Bod Pod for different waist and hip circumferences. The findings suggest that all three BIA models are accurate to estimate BF% when compared to the Bod Pod with less accuracy for those with greater waist and/or hip circumferences. This study adds to the literature as no research has attempted to test the accuracy of hand to hand, foot to foot, and hand to foot BIA against a gold standard measure in the same study. Also, most validation studies were completed with only one BIA model compared to a gold standard measurement [5–7].

Table 1 reports a difference in age among the tertiles of waist circumferences and a significant difference of BMI among the tertiles of hip circumferences; both of these variables have been reported to negatively impact the accuracy of BIA [8, 9, 12]. The accuracy of BIA models to estimate BF% in 2/3 of the groups (tertiles 1-2) can be explained by the rigorously controlled experiment in the current study. It is possible that if one did not follow the pretesting protocol, more variability would be observed in the sample with slimmer waist and/or hip.

Aldosky et al. (2018) also looked at the accuracy of BIA to characterize regional body fat distribution and its correlation with anthropometric measurements in a sample of women [10]. In their analysis, they demonstrated that foot to foot BIA was more accurate with subjects having a large hip circumference (r=0.942, p<0.001), while hand to hand BIA was more accurate with subjects having a large waist circumference (r=0.975, p<0.001). Even if they demonstrated a high correlation between anthropometric measures and the BIA measures, the BF% observed was not validated against a gold standard measure. It is possible that a high correlation would not be associated when testing the difference between the two measures and the average of the measures.

The gold standard measurements of body composition, the DXA scan and the Bod Pod, are not well suited for everyday use by the average person. They are very expensive and too large to keep at home and, in the case of the DXA, require a certified room and a trained operator. The three BIA devices that were used in this study were not significantly different with the Bod Pod with the exception of the highest tertiles of hip and waist for all BIA apparatus. For the highest tertile of waist and hip, the average difference was around 2.48% compared with the Bod Pod estimation. One could use the result given by a BIA apparatus and add 2.48% and be closer to the estimation of the Bod Pod. As a result, the BIA devices tested in this study could be used in some settings.

Strengths of this study include the use of Bod Pod to estimate BF%, a well-accepted measure as the criterion method in the interpretation of results between different BIA models. This study also included participants with a great range of age and hip and waist circumferences. In addition, this study is, to our knowledge, the first study to compare different BIA models based on different hip and waist circumferences among men and women against a gold standard measure to capture BF%, testing the accuracy of these practical tools when BF% changes over time or the accuracy of the BIA models when not respecting pretesting procedures. We have also only tested with our three models of BIA so these results should not be assumed to be accurate when using other models of BIA.

## 5. Conclusion

In conclusion, the three BIA models offer a BF% measurement agreeing with the estimation obtained with the Bod Pod with the exception of people having a greater waist or greater hip. Given the fact that many people are interested in knowing their BF%, and the small difference observed, even in the highest waist circumferences and/or hip circumferences (less than 3% difference on average) when pretesting conditions are respected, our results are relevant.

## Figures and Tables

**Table 1:** Participants' Characteristics.

	*Total N=92*	*Waist Circumference*			*Hip Circumference*		
Tertiles		*Tertile 1*	*Tertile 2*	*Tertile 3*	*Tertile 1*	*Tertile 2*	*Tertile 3*
Ranges (cm)		70.1-87.9	88.0-96.9	97.0-127	88.0-99.9	100.0-106.9	107.0-132.5
Age (years)	43.8 ±17.5	35.6±17.6	45.8±17.2	49.8± 15.0**∗**	42.7±17.9	44.9±17.9	43.6±17.2
Sex (Men)	51 (55.4%)	17 (56.7)	17 (54.8)	17 (54.8)	13 (43.3)	18 (56.3)	20 (66.7)
Body Mass Index (kg/m^2^)	25.5 ± 4.3	21.6±2.1	25.5±2.5	29.3±4.0**∗**	21.6±2.1	25.2±1.8	29.5±4.1**∗**
Waist circumference (cm)	92.2 ±11.3	80.7±4.7	91.4±2.6	104.6±8.7**∗**	83.3±7.0	91.6±6.9	101.4±11.2**∗**
Hip circumference (cm)	103.4 ± 8.6	96.8±5.3	103.6±6.9	109.7±8.2**∗**	94.8±3.1	102.1±1.7	112.9±6.5**∗**

Data presented as average **± **SD or N (%); *∗*P<0.05 among tertiles

## Data Availability

The data used to support the findings of this study are available from the corresponding author upon request.
